# Patterns of service use in a culturally adapted NAVIGATE program for first-episode psychosis and their association with clinical and functional outcomes

**DOI:** 10.1192/j.eurpsy.2025.696

**Published:** 2025-08-26

**Authors:** G. Hoter Ishay, D. Roe

**Affiliations:** 1 Occupational Therapy, Ono academic college, Kiryat Ono; 2 Community Mental Health, University of Haifa, Haifa, Israel

## Abstract

**Introduction:**

NAVIGATE, is a comprehensive manual based intervention developed in the US for young people experiencing a first episode psychosis (FEP). The intervention is based on four main service components: Individual Resilience Training (IRT), supported employment and education (SEE), family psychoeducation (FEP) and medication management.

**Objectives:**

To describe the process of implementing NAVIGATE in Israel with an emphasis on cultural adaptation, fidelity, patterns of use and their relation to outcomes.

**Methods:**

Between 2017-2021 demographic and diagnostic, service utilization data and ratings of functioning and symptoms were collected from clinical registries of 142 NAVIGATE participants.

**Results:**

Most participants were males (70%), aged 23.5 (SD 5.5, Range 16-43). On average, participated in the program over a year. IRT was the most utilized intervention (M=23, SD 11.51). Overall, three clusters of program usage were found. Number of sessions and their frequency were highly correlated. Number of family psychoeducation meetings showed the highest correlation with improvement overtime in functioning and symptoms severity. Program Fidelity rates ranged from 2.8 to 3.3 (range 0-4).

**Conclusions:**

The NAVIGATE program in Israel demonstrated significant clinical and functional outcomes across all service use patterns. NAVIGATE was formally offered for two years and included four components, in reality, service users attended less than what was offered. It is possible that when an effective comprehensive team-based intervention is offered flexibly to meet the often rapid changes needs of young people with FEP, the actual use is less than one might expect, which has important implications for policy and practice.

**Disclosure of Interest:**

None Declared

